# Sonodynamic Therapy for Gliomas. Perspectives and Prospects of Selective Sonosensitization of Glioma Cells

**DOI:** 10.3390/cells8111428

**Published:** 2019-11-13

**Authors:** Krzysztof Bilmin, Tamara Kujawska, Paweł Grieb

**Affiliations:** 1 Department of Experimental Pharmacology, Mossakowski Medical Research Centre, Polish Academy of Sciences, 02-106 Warsaw, Poland; pgrieb@imdik.pan.pl; 2 Faculty of Medicine, Collegium Medicum, Cardinal Stefan Wyszynski University, 01-938 Warsaw, Poland; 3 Institute of Fundamental Technological Research, Polish Academy of Sciences, 02-106 Warsaw, Poland; tkujaw@ippt.pan.pl

**Keywords:** glioma, ultrasound, sonodynamic therapy, ALA

## Abstract

Malignant glial tumors (gliomas) are the second (after cerebral stroke) cause of death from diseases of the central nervous system. The current routine therapy, involving a combination of tumor resection, radio-, and chemo-therapy, only modestly improves survival. Sonodynamic therapy (SDT) has been broadly defined as a synergistic effect of sonication applied in combination with substances referred to as “sonosensitizers”. The current review focuses on the possibility of the use of tumor-seeking sonosensitizers, in particular 5-aminolevulinic acid, to control recurring gliomas. In this application, SDT employs a principle similar to that of the more widely-known photodynamic therapy of superficially located cancers, the difference being the use of ultrasound instead of light to deliver the energy necessary to eliminate the sensitized malignant cells. The ability of ultrasound to penetrate brain tissues makes it possible to reach deeply localized intracranial tumors such as gliomas. The major potential advantage of this variant of SDT is its relative non-invasiveness and possibility of repeated application. Until now, there have been no clinical data regarding the efficacy and safety of such treatment for malignant gliomas, but the preclinical data are encouraging.

## 1. Introduction

Malignant glial tumors (gliomas) are the second (after cerebral stroke) greatest cause of death from diseases of the central nervous system. Glioblastoma multiforme (GBM), the commonest form of glioma (ca. 50% of cases), is one of the most aggressive malignant tumors developing in adult patients. Although the incidence of GBM increases with age [1], almost one half of cases are diagnosed in patients aged 40–64 years [2]. The effective therapy of GBM is an unmet medical need, because those currently applied, usually consisting of a combination of surgical intervention (tumor resection), radiotherapy, and chemotherapy, do not offer satisfactory effects. Currently, the standard of care in the pharmacotherapy of infiltrating gliomas is temozolomide—an alkylating compound administered orally, used in combination with radiotherapy or as monotherapy. Additionally, in the Unites States, the Food and Drug Administration (FDA) has approved bevacizumab—an agent that blocks the development of pathological angiogenesis via inhibition of vascular endothelial growth factor (VEGF) [3]. Nevertheless, regardless of the treatment scheme, the median survival in GBM is 1–1.5 years [4], and the five-year survival rate is less than 5% [5,6].

The unsatisfactory efficacy of GBM treatment results from the chemo- and radio-resistance of the self-renewing and fast-proliferating tumor stem cells (TSCs), which infiltrate healthy brain tissues, crawling along blood vessels, and the axonal pathways are large distances from the glioma foci and are responsible for recurrence [7]. The additional feature of malignant gliomas that accounts for the low efficacy of chemotherapy is the blood–brain barrier (BBB). Even if levels of cytotoxic drugs that are sufficient to kill malignant cells are achieved in parts of the tumors, visible in contrast-enhanced NMR images where the barrier is damaged, the drug concentrations are probably several times lower in the peritumoral zone where the TSCs reside, surrounded by an intact barrier (such a phenomenon was found in the brain metastases [8]).

The aforementioned attributes of the malignant gliomas result in that, independent of the completeness of the surgical removal of the lesion, tumors always recur. The issue of re-operating recurrent GBM is controversial [8], but a recent meta-analysis shows that repeat surgery prolongs the survival of some patients with recurrent GBM, independently of other prognostic factors [9]. However, neurosurgical resection cannot be repeated many times. Further progress may be achieved by using newly developed technologies, enabling the elimination of glioma foci. For example, promising clinical results have been obtained with stereotactic radiosurgery, which delivers high-dose radiation on the recurred tumor bed, while sparing the adjacent normal brain tissues, and may be applied alone or in combination with chemotherapy [10]. The other example is MRI-guided laser interstitial thermal therapy, a minimally invasive neurosurgical technique of tumor cytoreduction recently tested in the clinic, which also disrupts BBB for a period of 4–6 weeks [11]. However, in these approaches, the precondition of therapeutic success is that all tumor cells are localized prior to the delivery of focused therapy. In the case of malignant gliomas, this requirement is practically never met.

Sonodynamic therapy (SDT) has been defined as the application of non-thermal ultrasound energy for the treatment of diseases in combination with drugs [12]. Only two reviews have been published to date dedicated specifically to SDT and gliomas [13,14]; moreover, they both discuss various applications of this technique (e.g., for opening BBB or otherwise increasing drug delivery to the tumor cells, enhancing immunotherapy, etc.). The current review is more focused, it discusses only the application of low intensity ultrasound, following sonosensitization by tumor-seeking agents related to porphyrins, to eliminate glioma cells and control glioma recurrence. This type of SDT is basically similar to the intraoperative photosensitization of glioma cells prior to their surgical removal, more widely known and currently approved for clinical use in several countries, the difference being the use of ultrasound instead of light to deliver energy to sensitized malignant cells [15,16]. To facilitate understanding the specificity and potential advantages of this type of SDT for the treatment of gliomas, we start with a short presentation on ultrasound (US), its interactions with tissues, and its applications in medicine.

## 2. Ultrasound and Its Medical Applications

Ultrasound (US), or ultrasonic waves, are mechanical vibrations of elastic media, with frequencies of over 16 kHz, which is above the range of human hearing. Ultrasonic waves propagating in soft tissues and similar elastic media are being used for many purposes in medicine. Medical diagnostics use waves with frequencies ranging from several or several dozens of MHz (for examination of soft tissues) to 100 MHz (for examination of skin and eyes), and physical therapy uses waves with lower frequencies (from 750 kHz to 3 MHz) [17,18,19]. Low intensity ultrasonic waves have numerous applications in medicine. Their usefulness in medical diagnostics is associated with the ability to penetrate soft tissues and to reflect from the borders of tissue structures, presenting different acoustic impedances (elasticity). The received echo-signals are transformed into grey-scale images, providing information regarding the anatomical structure of tissues, any changes of that structure, or movement. An ultrasonic image is a product of the transformation of echo-signals received with a probe from the immobile tissue structures of various impedance (ultrasonography or medical ultrasound) or from mobile structures (e.g., flowing blood) with a Doppler probe, using the change of frequency between the emitted and received signals (the Doppler effect). The second favorable property of low-intensity ultrasonic waves is their harmlessness, which allows for repeatable applications, for instance as a diagnostic imaging technique. Currently, ultrasound imaging and Doppler sonography have become routine diagnostic procedures in various fields of medicine (e.g., in obstetrics).

The use of ultrasound for therapeutic purposes usually consists of the induction of irreversible biological effects. The range of biological effects depends on the local intensity of the ultrasound beam, and on the time of exposure. The therapeutic effects of the focused ultrasound depend largely on the ability of a treated tissue to absorb the acoustic energy. Energy absorption leads to the emission of heat. Lately, growing interest is being observed in the applicability of ultrasound to the treatment of neurodegenerative diseases and tumors [19]. An example of therapeutically beneficial high intensity focused ultrasound (HIFU) is the selective heating of a tumor localized deep under the skin. This technique is used, among others, for the low-invasive treatment of uterine fibroids [20].

There are two kinds of ultrasound effects on biological systems. The thermal effect is based on the fact that locally accumulated acoustic energy is absorbed and transformed into heat. The thermal effect depends both on the level of local intensity of the ultrasonic beam used, as well as on the level of absorption and the dispersion of acoustic waves in the tissues examined, and the time of sonication. The propagation of ultrasonic waves in tissues can also be associated with non-thermal effects, such as cavitation and shear forces in cellular membranes.

### 2.1. The Thermal Mechanism

In the process of interference with a biological system, the acoustic energy of an ultrasound is absorbed and transformed into heat. The resulting growth of temperature depends on both the acoustic parameters of the ultrasonic beam (its local intensity, frequency, and exposure time) and on the acoustic and thermal properties of the tested biological medium (its thermal conductivity, diffusiveness, and specific heat [21]). In some therapeutic applications of ultrasound, the desired effect involves a non-invasive heating of a small, local volume within a tissue. The efficacy of that treatment depends on the ability to achieve the desired growth of temperature. The produced heat may, for example, increase the kinetic energy of the components of the cellular membrane (proteins, lipids, and carbohydrates), and increase its permeability. In some cases, the growth of temperature may lead to a damaging of the cells, and even to their rapid death [22]. Experiments on local hyperthermia in animal models indicate that the level of thermal cellular damage depends mostly on the rise of temperature, exposure time, and susceptibility of tissues [23,24].

### 2.2. Tension Mechanisms

Tensions may appear in the biological systems exposed to ultrasound, as a result of the action of variable forces caused by the variable pressure of acoustic waves on the biological membranes. The level of those tensions and their induced biological effects depend on the acoustic properties of the ultrasound field. and on the acoustic and thermal properties of the biological system used. For example, the effects in in vitro cellular systems may be caused by mechanisms acting both inside and outside the cellular membrane, activating a tension mechanism. Ultrasound waves propagating in cellular systems may cause the torsion or rotation of cells. Tensions associated with the micro-stream flow may cause a change of strain on the surface of a membrane, the transient formation of pores, or may increase its permeability [25,26].

### 2.3. Cavitation Mechanisms

Cavitation is a physical phenomenon involving the formation of micro-bubbles filled with vacuum, saturated steam, or gas in a liquid medium or tissue. Under the influence of ultrasonic waves of varying pressure falling on them, the bubbles begin to oscillate and crack, which can lead to local disruptions in the structure of the medium. Cavitation is a very important mechanism of interaction of ultrasound with biological media. The condition for the appearance of cavitation is reaching or exceeding the, so-called, cavitation threshold (i.e., the threshold value of ultrasound intensity). This value depends on the temperature of the medium, its aeration or gas content, and the frequency of the ultrasonic waves. The cavitation processes are divided into non-inertial cavitation (constant) and inertial cavitation (transient).

In the case of non-inertial cavitation, oscillating micro-bubbles may cause cell membrane vibrations, leading to stream flows both inside and outside the cell. As a result, the membrane is subjected to shear stresses, which may cause micro-stream flows [27] inside and outside the cell, changes in the position of the intracellular organelles, or transient disruptions in the membrane’s structure or its damage [28].

Inertial cavitation occurs at high intensities of ultrasound. Cavitational bubbles, oscillating as a result of the non-linearly changing pressure in ultrasonic pulses, can increase their diameter even several times (during the compression phase), and then collapse rapidly (during the wave decompression phase) under the influence of negative pressure. This process is accompanied by the release of a large dose of energy, which has to be dissipated in a very small volume. This leads to a local increase in pressure and temperature. A rapid drop in pressure inside the bubble leads to its rupture and to the formation of a shock wave, destroying the adjacent biological structures. Such pressure–temperature conditions contribute to the disintegration of water molecules, resulting in the formation of hydrogen atoms and hydroxyl radicals [29,30].

## 3. Sonodynamic Therapy of Cancers: Definitions and Mechanisms

In the field-defining review published in 2004, Rosenthal et al. [28] described sonodynamic therapy (SDT) as an approach based on the synergistic effect of ultrasound and a chemical compound referred to as a “sonosensitizer”. Such a broad definition covers two meanings of the term “sonosensitization”. The first is the use of ultrasound energy to enhance the action of drugs displaying their own therapeutic activity, for example, by increasing the penetration of cytotoxic drugs to cancer cells (such action of ultrasound could perhaps be called “chemosensitization”). The other meaning of “sonosensitization” is the use of substances—let us call them “true” sonosensitizers—which, by themselves are not active, to sensitize cells to ultrasound. This meaning would be analogous to “radiosensitization”, the use of substances that are not active themselves, but sensitize cells to radiation. The aforementioned review [28] mostly concerned oncological applications of SDT, aimed at the elimination of cancer cells. The following three types of substances were listed as sonosensitizers potentially useful for cancer therapy: anticancer cytotoxic drugs (e.g., anthracyclines), porphyrins (e.g., hematoporphyrin), and other substances (e.g., some anti-inflammatory drugs and dyes). In a more recent review concerning the SDT of cancers (McHale et al., 2016), the definition of sonodynamic therapy was narrowed down to the sensitization of target tissues with a non-toxic sensitizing chemical agent, prior to the exposure of the sensitized tissues to relatively low-intensity ultrasound [31]. The mechanisms involved in the SDT of tumors are not fully understood. However, some hypotheses have been proposed in the literature.

The first one assumes that the reactive oxygen species—singlet oxygen—is the active substance induced in the process of sonodynamic reactions, leading to cellular death [32].

The other hypothesis suggests that sonosensitizing is a result of a chemical activation of a sensitizer located in the vicinity of breaking the cavitation bubbles. A reaction with a hydroxyl radical or with a hydrogen atom would then lead to the formation of a radical on the sensitizer. The newly formed radical reacts with oxygen, forming superoxide and alkoxy radicals characterized by a long life and high mobility over large distances [33].

The third hypothesis assumes that sono-mechanical changes lead to the destruction of cells. The authors of that hypothesis suggest that the sensitizer causes the destabilization of the cellular membrane. As a result, a cell becomes susceptible to micro-flows, caused by the oscillations and fast movement of bubbles that injure cells on their way [28].

The fourth hypothesis assumes that the ultrasound wave leads to the formation of cavitation bubbles. Breaking bubbles cause rapid changes of pressure in the medium, which may lead to the emission of light. The light wave occurring in that process may activate the sonosensitizer. The phenomenon is called sonoluminescence [34].

Another hypothesis made by Tata [35] says that cavitation plays the main role in the increased production of OH radicals in the presence of anti-cancer drugs.

Among the three types of sonosensitizers listed in the aforementioned review published in 2004, porphyrins appeared to be particularly interesting, because of their tumor-localizing properties, which have been traditionally used in photodynamic diagnostics and therapy (PDD and PDT). The newer review of the subject [31] limited the list of sonosensitizers to non-toxic substances and dyes, mostly those used in PDD/PDT, and introduced 5-aminolevulinic acid (ALA), an amino acid precursor of protoporphyrin IX. Considering the ability of the focused ultrasound to penetrate tissues and to accumulate the acoustic energy in a small volume inside a tissue, the SDT seems to be a potentially superior option, particularly in case of hard to reach and deeply localized tumors, such as gliomas. Moreover, if a sono-sensitizing agent will be selectively accumulated in cancer cells, the SDT may prove safer, with no harmful effect on healthy brain cells.

**Figure 1:**
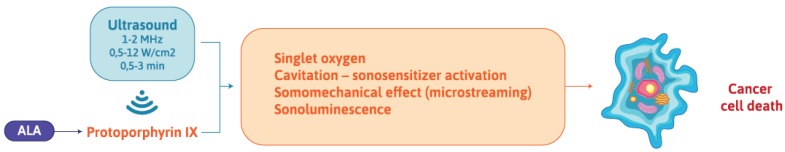
Diagram of the aminolevulinic acid (ALA)-mediated sonodynamic effect mechanism in a cancer cell.

The scheme and main mechanisms of the sonodynamic effect are illustrated in Figure 1.

Compared with the laser beam used in photodynamic therapy for years, ultrasound more easily penetrates the body and allows for the local activation of an administered drug within a small volume of a tumor, thus limiting the injuries of the surrounding, healthy tissues. The first report of the SDT was published in 1990. Umemura et al. [36] described the synergistic effect of ultrasound waves and hematoporphyrin on an in vitro murine model of sarcoma. The authors demonstrated that the cellular injury was caused by the formation of singlet oxygen from hematoporphyrin activated by ultrasound.

## 4. 5-Aminolevulinic acid (ALA): A Tumor-Seeking Agent Useful for the Photodynamic Detection of Gliomas

**Figure 2:**
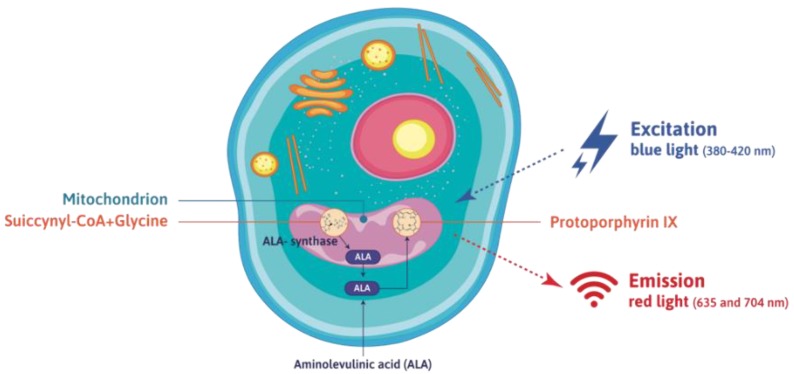
Synthesis of aminolevulinic acid (ALA) in the cell and mechanism of the photodynamic effect—activation of protoporphyrin IX with blue light and the emission of red light.

ALA, amino acid precursor in the biosynthesis of heme, is formed in the mitochondria from glycine and succinyl-CoA in the reaction catalyzed by ALA synthase. The one before last metabolite in the pathway of heme synthesis is the protoporphyrin IX (PpIX), demonstrating photo-sensitizing properties (see Figure 2). Administration of ALA increases the synthesis of PpIX and leads to its accumulation in proliferating cells - mostly cancer cells in the brain of a patient with glioma. Such a phenomenon is the consequence of different activity of enzymes involved in PpIX synthesis in cancer and normal cells. In particular, the activity of ferrochelatase, the enzyme which catalyses insertion of the ferrous ion to PpIX, is reduced in cancer cells [37].

The photo-active PpIX excited with blue light of 380–420 nm wavelength emits red light of 635 and 704 nm wavelengths. This property of protoporphyrin is used in neurosurgery for fluorescent-guided resections [37,38,39]. Prior to the tumor resection surgery, three hours before the administration of anesthesia, a patient is given ALA at an oral dose of 20 mg per kg of body weight. During the surgical procedure, the surgical microscope equipped with appropriate filters reveals that cancer cells excited with blue light emit red (bulk of the tumor tissue) or pink (infiltration of healthy cerebral tissues with glioma cells) light, while healthy cells are visible as blue. The method allows for a more complete resection of the tumor, as the surgeon is able to precisely remove both the bulk tumor tissue and some glial cells infiltrating the surrounding healthy tissues [24,39].

Studies indicate that an increased range of resection of glioma results in an increased survival of patients. As mentioned above, the fluorescent detection of cancer cells with aminolevulinic acid (ALA) is used in order to increase the efficacy of resection. However, considering the infiltratory character of the growth of gliomas, a complete elimination of cancer cells is difficult. In particular, problems with precise resection may occur because of the low efficiency of ALA accumulation in glioma cancer stem cells (GSC). In a study of rat glioma C6, it was found that GSCs, which are very aggressive when implanted into immunodeficient mice, exhibit a low PpIX accumulation. The authors suggested that these subpopulations of cancer cells may not exhibit enough fluorescence during 5-ALA-guided surgery to allow for their removal, which consequently will lead to an incomplete resection and recurrence of the disease. However, they were able to increase the accumulation of PpIX in GSC by deferoxamine (iron chelator), which caused a decrease in the concentration of iron ions. The authors proposed that this approach can be quickly introduced into clinical practice [40].

At present, in neurosurgery, ALA is routinely used only for intrasurgical navigation, although there are also some data available regarding the use of the compound as a photo-sensitizing agent in the photo-dynamic therapy. In a small clinical trial, patients with glioblastoma multiforme were administered ALA, together with the photo-sensitizing porphyrin derivative Photofrin (porfimer sodium), as a support for the surgical resection of tumors. After that, for three days surgical beds were repeatedly (four times) irradiated with light from a diode laser, to be absorbed by the glioma cells remaining in the patient’s brain and sensitized by the photo-sensitizers. In the group of patients treated with the PDD and PDT, the mean survival time was 52.8 weeks, compared to the control group subjected to conventional surgery, with the mean survival time of just 24.6 weeks [41]. The result was impressive, nonetheless all of the gliomas recurred.

## 5. ALA and Porphyrins in Pre-Clinical Models of Glioma: Research Review

Recently, significant progress has been observed in the development of SDT. Studies indicate that the SDT may be a promising therapeutic strategy for many types of cancer (breast, liver, stomach, and prostate) [34]. However, gliomas are more difficult to treat, being deeply located inside the skull and deeply infiltrating the healthy brain tissues.

Research on the use of sonosensitizers and ultrasound for the sonodynamic treatment of gliomas is in the very early stage. However, the preliminary data published to date seem to open a fascinating perspective of a new modality for glioma treatment, which could be selective to malignant cells—therefore not requiring prior localization malignant cells, and at the same time virtually noninvasive—therefore enabling its repeated application.

### 5.1. In Vitro Studies

Endo et al. studied the effect of SDT with ALA, PpIX, and talaporfin on human glioma U87MG, and on rat glioma C6. The cytotoxic effect of SDT was observed only for rat glioma, which was associated with an increased accumulation of the sono-sensitizer in rat cells, compared with the human ones. A similar efficacy was demonstrated for the SDT combined with talaporfin and ALA, and it was higher than that for PpIX. Also, a higher number of apoptotic cells was noted both for the treatment involving ultrasound alone and combined with ALA in the cell line C6 [42]. The apoptosis in the C6 glioma cells was also induced by the SDT with hematoporphyrin monomethyl ether (HMME). The key role in the induction of apoptosis was played by oxygen free radicals, the loss of the mitochondrial membrane potential, and the increase of the cellular Ca^2+^ ion level [43]. An increased level of pro-apoptotic proteins, such as, caspase 3 and 9, Bax, Bcl-2, and Fas-L, was also found [44]. Some interesting results were published by Xu et al. The authors concluded that glioma stem-like cells (GSCs) were less susceptible to SDT. Based on the experiment on the cell line of human glioma U251, a lower concentration of the sono-sensitizer (porfimer sodium) and a reduced production of reactive oxygen species (ROS) in the GSCs were observed, compared with the glioma U251 cell line. This is as a result of the fact that there is an increased amount of the ABCG2 protein—a membranous pump with the ability to actively remove the drugs from the cell—on the surface of the cancer stem-like cells [45]. However, the use of the ABCG2 inhibitor (fumitremorgin C) caused an increase of the intracellular content of the sono-sensitizer and accounted for the increased production of ROS and increased effectiveness of the SDT [46]. The synergistic anti-tumor effect of the SDT with ALA and hyperthermia therapy was also observed in the human glioma cells in the U87MG cells in vitro, and it turned out that a combination of SDT and hyperthermia therapy gave a higher cytotoxic effect than SDT alone, and induced a higher amount of ROS and pro-apoptotic proteins [47]. In recently published work, a similar mechanism of action was described in a study on ALA-mediated SDT in U87 and U251 glioblastoma cell lines, and apoptosis induction and increased ROS production was observed [48]. In our previous study, we also observed a sonodynamic effect using 5-ALA in the rat glioma RG2 cell line. The biggest cytotoxic effect was observed for the combination 5-ALA and 6 W ultrasound power [49].

### 5.2. In Vivo Studies

**Figure 3:**
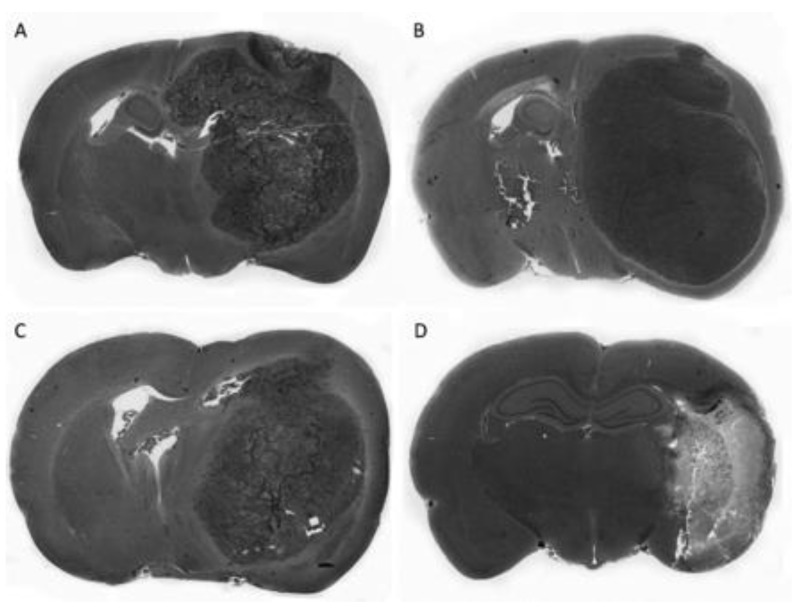
Pivotal study of 5-aminolevulinic acid (ALA)-mediated sonodynamic effect on a rat C6 glioma model in vivo. A significant reduction of the tumor mass after sonodynamic therapy is shown (**C**). The other photos show the control groups, namely: untreated (**A**), 5-ALA only (**B**), and ultrasound only (**D**). This figure has been reproduced from the literature [50], with permission from the publisher.

The effectiveness of the SDT was also assessed in *in vivo* animal models. Ohmura et al. assessed the efficacy of the SDT with ALA on the rat model of glioma C6. The authors studied also the effect of ultrasound alone, applied with intensities of 10 and 15 W/cm^2^ to healthy brain tissue. A massive loss of the cerebral tissue was notable after the application of ultrasound, with a frequency of 1.04 MHz and a local intensity of 15 W/cm^2^ for 5 min of exposure. For that reason, ultrasound of a lower intensity (10 W/cm^2^), as well as the same frequency of 1.04 MHz and the exposure time (5 min), were used in the SDT. A significant reduction of tumor size compared to the control group and to the group exposed to ultrasound alone was observed in the brains of the animals treated with SDT (Figure 3) [50]. In another, similar study of the efficacy of the SDT with ALA, performed on the same animal model, different acoustic parameters of ultrasonic waves were used (intensity, 2.65 W/cm^2^; frequency, 1.0 MHz; exposure time, 20 min). Two weeks after the application of SDT in the control group, the mean volume of the tumor was 122.48 ± 39.64 mm^3^, in the group treated with ultrasound alone 87.42 ± 21.40 mm^3^, and in the group receiving the SDT with ALA only 10.50 ± 8.20 mm^3^ [51]. In an experiment on a rat model of glioblastoma C6 implanted in Sprague Dawley rats, a good response to the therapy and an increased survival rate was observed in the group treated with ALA-mediated SDT. Such an effect was not observed in the groups of animals receiving only ALA or ultrasound alone. The authors also studied a very important parameter potentially limiting the use of SDT, namely, the thermal effects generated by ultrasound. The 5.5 W ultrasound power determined during this experiment generated a slight temperature rise by about 2 °C. That acoustic power of the ultrasound was considered as safe and was used in SDT [52].

The aforementioned data are important and interesting, but further studies are certainly necessary. In particular, the confirmation of the sonodynamic effect in other pre-clinical models of glioma, besides the most commonly used C6 rat glioma cell line, is required. Further studies are also needed in order to determine the optimal parameters of the ultrasonic waves required to induce the sonodynamic effect in malignant cells, while not causing damage to the healthy brain tissues. The results obtained in such experiments could be invaluable for planning further preclinical *in vivo* studies, and in the future, also clinical trials.

## References

[1] Eder K., Kalman B. (2014). Molecular heterogeneity of glioblastoma and its clinical relevance. Pathol. Oncol. Res..

[2] Li K., Lu D., Guo Y., Wang C., Liu X., Liu Y., Liu D. (2018). Trends and patterns of incidence of diffuse glioma in adults in the United States, 1973-2014. Cancer Med..

[3] Lukas R.V., Mrugala M.M. (2017). Pivotal therapeutic trials for infiltrating gliomas and how they affect clinical practice. Neurooncol. Pract..

[4] Hervey Jumper S.L., Berger M.S. (2016). Maximizing safe resection of low- and high-grade glioma. J. Neurooncol..

[5] Ostrom Q.T., Bauchet L., Davis F.G., Deltour I., Fisher J.L., Langer C.E., Pekmezci M., Schwartzbaum J.A., Turner M.C., Walsh K.M. (2015). The epidemiology of glioma in adults: A “state of the science” review. Neuro. Oncol..

[6] Thakkar J.P., Dolecek T.A., Horbinski C., Ostrom Q.T., Lightner D.D., Barnholtz-Sloan J.S., Villano J.L. (2014). Epidemiologic and molecular prognostic review of glioblastoma. Cancer Epidemiol. Biomark. Prev..

[7] Bradshaw A., Wickremsekera A., Tan S.T., Peng L., Davis P.F., Itinteang T. (2016). Cancer Stem Cell Hierarchy in Glioblastoma Multiforme. Front. Surg..

[8] Robin A.M., Lee I., Kalkanis S.N. (2017). Reoperation for Recurrent Glioblastoma Multiforme. Neurosurg. Clin. N. Am..

[9] Lu V.M., Jue T.R., McDonald K.L., Rovin R.A. (2018). The Survival Effect of Repeat Surgery at Glioblastoma Recurrence and its Trend: A Systematic Review and Meta-Analysis. World Neurosurg..

[10] Cheon Y.J., Jung T.Y., Jung S., Kim I.Y., Moon K.S., Lim S.H. (2018). Efficacy of Gamma Knife Radiosurgery for Recurrent High-Grade Gliomas with Limited Tumor Volume. J. Korean Neurosurg. Soc..

[11] Leuthardt E.C., Duan C., Kim M.J., Campian J.L., Kim A.H., Miller-Thomas M.M., Shimony J.S., Tran D.D. (2016). Hyperthermic Laser Ablation of Recurrent Glioblastoma Leads to Temporary Disruption of the Peritumoral Blood Brain Barrier. PLoS ONE.

[12] Tachibana K., Feril L.B., Ikeda-Dantsuji Y. (2008). Sonodynamic therapy. Ultrasonics.

[13] Hersh D.S., Kim A.J., Winkles J.A., Eisenberg H.M., Woodworth G.F., Frenkel V. (2016). Emerging Applications of Therapeutic Ultrasound in Neuro-oncology: Moving Beyond Tumor Ablation. Neurosurgery.

[14] Wang X., Jia Y., Wang P., Liu Q., Zheng H. (2017). Current status and future perspectives of sonodynamic therapy in glioma treatment. Ultrason Sonochem..

[15] Akimoto J. (2016). Photodynamic Therapy for Malignant Brain Tumors. Neurol. Med. Chir. (Tokyo).

[16] Lakomkin N., Hadjipanayis C.G. (2018). Fluorescence-guided surgery for high-grade gliomas. J. Surg. Oncol..

[17] Miłkowska K. (2007). Ultrasound – mechanisms of action and application in sonodynamic therapy. Postepy Hig. Med. Dosw..

[18] Miller D.L., Smith N.B., Bailey M.R., Czarnota G.J., Hynynen K., Makin I.R. (2012). Overview of therapeutic ultrasound applications and safety considerations. J. Ultrasound Med. Biol..

[19] Robertson V.J., Ward A.R. (1990). Longwave ultrasound reviewed and reconsidered. Physio-Ther..

[20] Mahmoud M.Z., Alkhorayef M., Alzimami K.S., Aljuhani M.S., Sulieman A. (2014). High-Intensity Focused Ultrasound (HIFU) in uterine fibroid treatment: Review study. Pol. J. Radiol..

[21] Kujawska T., Secomski W., Kruglenko E., Krawczyk K., Nowicki A. (2014). Determination of tissue thermal conductivity by measuring and modeling temperature rise induced in tissue by pulsed focused ultrasound. PLOS One.

[22] ter Haar G. (1999). Therapeutic ultrasound. Eur. J. Ultrasound.

[23] Barnett S.B. (2000). Biophysical aspects of diagnostic ultrasound. Ultrasound Med. Biol..

[24] Kujawska T., Secomski W., Bilmin K., Nowicki A., Grieb P. (2016). Impact of thermal effects induced by ultrasound on viability of rat C6 glioma cells. Ultrasonics.

[25] Barnett S.B. (1998). Ultrasound. Other non-thermal mechanisms: Acoustic radiation force and streaming. Ultrasound Med. Biol..

[26] Duck F.A. (2000). Radiation stress and its bio-effects. European Committee for Medical Ultrasound Safety (ESMUS). Eur. J. Ultrasound.

[27] Miller M.W., Mille D.L., Brayman A.A. (1996). A review of in vitro bioeffects of inertial ultrasonic cavitation from a mechanistic perspective. Ultrasound Med. Biol..

[28] Rosenthal I., Sostaric J.Z., Riesz P. (2004). Sonodynamic therapy - a review of the synergistic effects of drugs and ultrasound. Ultrasonics Sonochem..

[29] Fuciarelli A.F., Sisk E.C., Thomas R.M., Miller D.L. (1995). Induction of base damage in DNA solutions by ultrasonic cavitation. Free Radic. Biol. Med..

[30] Riesz P., Kondo T. (1992). Free radical formation induced by ultrasound and its biological implications. Free Radic. Biol. Med..

[31] McHale A.P., Callan J.F., Nomikou N., Fowley C., Callan B. (2016). Sonodynamic Therapy: Concept, Mechanism and Application to Cancer Treatment. Adv Exp Med Biol..

[32] Liu X.H., Li S., Wang M., Dai Z.J. (2015). Current Status and Future Perspectives of Sonodynamic Therapy and Sonosensitizers. Asian Pac. J. Cancer Prev..

[33] Mišik V., Riesz P. (2000). Free radical intermediates in sonodynamic therapy. Ann. N. Y. Acad Sci.

[34] Costley D., Mc Ewan C., Fowley C., McHale A.P., Atchison J., Nomikou N., Callan J.F. (2015). Treating cancer with sonodynamic therapy: A review. Int. J. Hyperth..

[35] Tata D.B., Biglow J., Tritton T.R., Dunn F. (1996). Ultrasound-enhanced hydroxyl radical pro-duction from two clinically employed anticancer drugs, adriamycin and mitomycin C. Ultrason. Sonochem..

[36] Umemura S., Yumita N., Nishigaki R., Umemura K. (1990). Mechanism of cell damage by ultrasound in combination with hematoporphyrin. Jpn. J. Cancer Res..

[37] Grieb P. (2004). 5-Aminolevulinic acid (ALA) and its applications in neurosurgery. Neurol. Neurochir. Pol..

[38] Carlsson S.K., Brothers S.P., Wahlestedt C. (2014). Emerging treatment strategies for glioblastoma multiforme. EMBO Mol. Med..

[39] Mansouri A., Mansouri S., Hachem L.D., Klironomos G., Vogelbaum M.A., Bernstein M., Zadeh G. (2016). The role of 5-aminolevulinic acid in enhancing surgery for high-grade glioma, its current boundaries, and future perspectives: A systematic review. Cancer.

[40] Wang W., Tabu K., Hagiya Y., Sugiyama Y., Kokubu Y., Murota Y., Ogura S.I., Taga T. (2017). Enhancement of 5-aminolevulinic acid-based fluorescence detection of side population-defined glioma stem cells by iron chelation. Sci. Rep..

[41] Eljamel M.S., Goodman C., Moseley H. (2009). ALA and Photofrin fluorescence-guided resection and repetitive PDT in glioblastoma multiforme: A single centre Phase III randomised controlled trial. Lasers Med. Sci..

[42] Endo S., Kudo N., Yamaguchi S., Sumiyoshi K., Motegi H., Kobayashi H., Terasaka S., Houkin K. (2015). Porphyrin derivatives-mediated sonodynamic therapy for malignant gliomas in vitro. Ultrasound Med. Biol..

[43] Hao D., Song Y., Che Z., Liu Q. (2014). Calcium overload and in vitro apoptosis of the C6 glioma cells mediated by sonodynamic therapy (hematoporphyrin monomethyl ether and ultrasound). Cell Biochem. Biophys..

[44] Dai S., Hu S., Wu C. (2009). Apoptotic effect of sonodynamic therapy mediated by hematoporphyrin monomethyl ether on C6 glioma cells in vitro. Acta Neurochir..

[45] Xu Z.Y., Li X.Q., Chen S., Cheng Y., Deng J.M., Wang Z.G. (2012). Glioma stem-like cells are less susceptible than glioma cells to sonodynamic therapy with photofrin. Technol. Cancer Res. Treat..

[46] Xu Z.Y., Wang K., Li X.Q., Chen S., Deng J.M., Cheng Y., Wang Z.G. (2013). The ABCG2 transporter is a key molecular determinant of the efficacy of sonodynamic therapy with Photofrin in glioma stem-like cells. Ultrasonics.

[47] Ju D., Yamaguchi F., Zhan G., Higuchi T., Asakura T., Morita A., Orimo H., Hu S. (2016). Hyperthermotherapy enhances antitumor effect of 5-aminolevulinic acid-mediated sonodynamic therapy with activation of caspase-dependent apoptotic pathway in human glioma. Tumour Biol..

[48] Suehiro S., Ohnishi T., Yamashita D., Kohno S., Inoue A., Nishikawa M., Ohue S., Tanaka J., Kunieda T. (2018). Enhancement of antitumor activity by using 5-ALA-mediated sonodynamic therapy to induce apoptosis in malignant gliomas: Significance of high-intensity focused ultrasound on 5-ALA-SDT in a mouse glioma model. J. Neurosurg..

[49] Bilmin K., Kujawska T., Secomski W., Nowicki A., Grieb P. (2016). 5-Aminolevulinic acid-mediated sonosensitization of rat RG2 glioma cells in vitro. Folia Neuropathol..

[50] Ohmura T., Fukushima T., Shibaguchi H., Yoshizawa S., Inoue T., Kuroki M., Sasaki K., Umemura S. (2011). Sonodynamic therapy with 5-aminolevulinic acid and focused ultrasound for deep-seated intracranial glioma in rat. Anticancer Res..

[51] Jeong E.J., Seo S.J., Ahn Y.J., Choi K.H., Kim K.H., Kim J.K. (2012). Sonodynamically induced antitumor effects of 5-aminolevulinic acid and fractionated ultrasound irradiation in an orthotopic rat glioma model. Ultrasound Med. Biol..

[52] Wu S.K., Santos M.A., Marcus S.L., Hynynen K. (2019). MR-guided Focused Ultrasound Facilitates Sonodynamic Therapy with 5-Aminolevulinic Acid in a Rat Glioma Model. Sci. Rep..

